# The mechanism of apoliprotein A1 down-regulated by Hepatitis B virus

**DOI:** 10.1186/s12944-016-0232-5

**Published:** 2016-03-25

**Authors:** Yuanyuan Wang, Junli Hao, Xiaohong Liu, Hongxin Wang, Xin Zeng, Jing Yang, Lei Li, Xi Kuang, Tao Zhang

**Affiliations:** School of Biomedical Sciences, Chengdu Medical College, Sichuan, 610500 China; Department of Pharmacology, Key Laboratory of Drug Targeting and Drug Delivery Systems, West China School of Pharmacy, Sichuan University, Sichuan, 610041 China

**Keywords:** Apolipoprotein A1, Hepatitis B virus, Hypermethylation, 5-aza-dC

## Abstract

**Background:**

Hepatitis B virus (HBV) infection correlated with the development of cirrhosis, liver failure and hepatocellular carcinoma (HCC), poses a huge health burden on the global community. However, the pathogenesis of chronic hepatitis B (CHB) remains unclear. Apolipoprotein A1 (ApoA1) mainly secreted by hepatocytes, represents the major protein component of high-density lipoprotein. ApoA1 secretion may be disrupted by HBV infection. In this study, we mainly investigated the molecular mechanism of ApoA1 down regulated by HBV for revealing the pathogenesis of CHB.

**Methods:**

ApoA1 expression in livers of CHB patients as well as healthy controls were performed by Real-time PCR (RT-PCR) and Western blot. The serum ApoA1 levels were measured by Enzymed-linked immunosorbent assay (ELISA). Expression of ApoA1 mRNA and protein levels were performed by RT-PCR and Western blot in human hepatoma HepG2 cells and subline HepG2.2.15 cells. HBV expression construct, pHBV1.3 were transfected into HepG2, the changes of ApoA1 mRNA and protein expression were detected by RT-PCR and Western blot. To further study the mechanism of ApoA1 down regulation by HBV, 11 CpG islands in ApoA1 promotor were tested for DNA methylation status by MSP. HepG2.2.15 cell lines were treated with DNA methyltransferase inhibitor 5-aza-deoxycytidine (5-aza-dC), then, expression of ApoA1 mRNA and HBV particles in the supernatant, as well as ApoA1 protein levels were detected by RT-PCR and Western blot. Secretion of HBsAg and HBeAg in HepG2 cells cotransfected with pApoA1 and pHBV1.3 constructs was tested by ELISA. Meanwhile, secretion of HBsAg and HBeAg in the supernatant were quantified by ELISA in the HepG2.2.15 cells treated with 5-aza-dC plus ApoA1 siRNA.

**Results:**

Expression of ApoA1 mRNA and protein levels, as well as serum ApoA1 levels in CHB patients were decreased corresponding healthy controls in vivo. In addition, the expression of ApoA1 mRNA and protein levels were down regulated in HepG2.2.15 cells correponding HepG2 cells, 11 CpG islands in ApoA1 promoter were tested for methylation status by MSP in HepG2.2.15 cells compared to HepG2 cells, while two CpG islands were found hypermethylated. Expression of ApoA1 mRNA and protein levels were increased in HepG2.2.15 cells treated with DNA methyltransferase inhibitor 5-aza-dC. Furthermore, overexpression of ApoA1 can enhance HBV expression in HepG2 cells while the inhibitory effect of 5-aza-dC on HBV expression was completely abolished by blocking 5-aza-dC-induced up-regulation of ApoA1 using RNAi.

**Conclusions:**

Epigenetic silencing of ApoA1 gene expression by CpG island DNA hypermethylation induced by HBV may contribute to the pathogenesis of CHB.

## Background

Around 3 billion people wordwide were infected with Hepatitis B virus (HBV) which causes persistent live diseases. Chronic hepatitis B (CHB) correlated with a significant increased risk of cirrhosis, liver failure and hepatocellular carcinoma (HCC), poses a huge health burden on the global community [1–3]. However, the pathogenesis of CHB remains unclear.

Apoliprotein A1 (ApoA1), as a major protein component of high-density lipoprotein (HDL) is secreted normally by live and intestine. It is well known that ApoA1 and HDL show besides the cholesterol transport from peripheral cells to the liver [4], also have the important anti-inflammatory properties [4–6]. Moreover, Hyperlipidemia and Atherogenesis are often accompanied by abnormal expression of ApoA1 and HDL [7, 8].

In previous studies, plasma ApoA1 levels were apparently decreased in CHB patients corresponding healthy controls in vivo [9]. Similar results had shown the expression of ApoA1 was significantly decreased in hepatoma HepG2.2.15 (integrated the HBV genome) compared to HepG2 cells in vitro. Moreover, ApoA1 expression and promoter activities were down-regulated by HBV in a dose dependent manner [10]. HBV mRNAs and ApoA1 mRNA had shown a negative correlation in two hepatoma cell lines [11]. Notably, our recent study showed HBx interation led to an inpaired lipid-binding ability of ApoA1 while ApoA1 overexpression inversely promoted HBV expression [12]. Taken together, ApoA1 was suppressed by HBV at least partly due to trancriptional suppression. However, the regulatory mechanism are almost unknown. Given the importance of ApoA1 in the cholesterol transport and HBV regulation [13], the aim of the present study was to investigate the molecular mechanism of ApoA1 suppression by HBV for revealing the pathogenesis of CHB.

## Patients and methods

### Human specimens and serum samples

Liver sections from 200 CHB patients and 50 healthy controls were collected for detection of ApoA1 protein levels in liver. Serum samples were collected from 250 CHB patients and 50 healthy controls for detection of circulating ApoA1 levels. CHB patients, defined as serum HBsAg positivity for at least 6 months, may have exhibited symptoms of hepatitis or abnormal hepatic function. All the patients were hospitalized in West China Hospital, Sichuan University for Infectious Diseases from January 2014 and December 2014 and provided the informed consent in written form. The study protocols were approved by the Ethics Committee of West China Hospital, Sichuan University.

## Methods

### Reagents and antibodies

DNA methyltransferase inhibitor 5-aza-deoxycytidine (5-aza-dC) (A3656) was purchased from Sigama; ApoA1 ELISA detection kit (ab108804) was purchased from Abcam. Superscript™ RT reagent kit (DRR037A, Takara BioInc., Shiga, Japan); ApoA1 specific siRNA and non-specific control (sc-63361, sc-37007, Santa Cruz Biotechnology); the rabbit anti-human ApoA1 (sc-30089, Santa Cruz Biotechnology); the mouse anti-human actin and the horseradish peroxidase-conjugated secondary antibodies (Zhongshan Goldenbridge Biotechnology, China); and the ECL-Plus chemiluminescence system (Applygen Technologies, Beijing, China).

### Cell culture and constructs

Human hepatocellular carcinoma HepG2 and HepG2.2.15 cell lines were obtained from the ATCC (Rockville, MD), HepG2.2.15 with abilities to produce HBV virus stably is derived from HepG2 cell lines [14]. HBV replication plasmid pHBV1.3, containing 1.3 copies of the HBV genome (D genotype), pCDNA3.1-ApoA1 (pApoA1), containing coding sequence of ApoA1 were both kept in lab [2, 12].

### Immunoblot analysis

An equal amount of protein from liver sections of CHB patients as well as healthy controls were separated in a 10 % SDS–polyacrylamide gel and probed with an anti-ApoA1 and an anti-human actin antibody. The bound antibodies were visualized with appropriate HRP-conjugated secondary antibodies using an ECL detection kit.

### RNA, DNA extraction and real-time PCR

Total RNA was extracted from HepG2.2.15 cells treated with 5-aza-dC or negative control by Trizol Reagent (Invitrogen), as recommended by the manufacturer. GAPDH was used as an internal standard for the quantification of real-time PCR (RT-PCR), the primers used to detect ApoA1 and GAPDH have been described in previous studies [2, 15].

Supernatant in HepG2.2.15 cells was harvested at 48 h after treatment with 5 μM 5-aza-dC or negative control for detection of HBV particles [16]. HBV particles were quantitatively measured by RT-PCR according to the manufacturer's instructions of HBV PCR assay II kit (Tiangen. Co. Ltd.).

### Enzymed-linked immunosorbent assay (ELISA)

ELISA tests for detection of ApoA1 expression in serum of CHB patients and healthy controls were performed as recommended by the manufacturer.

### Methylmion Specific PCR (MSP)

The methylation levels of ApoA1 promoter were detected by MSP in Shanghai Integrated Biotech Solutions Co., Ltd.

### Statistical analysis

Each experiment was performed in triplicate, and all experiments were repeated three times. The results are presented as the mean ± standard deviation. Using Student’s *t*-test for comparison between groups, *p value <0.05 and **p < 0.01 were considered as a significant difference.

## Results

### Expression of ApoA1 mRNA and protein levels was significantly decreased in CHB patients

**Fig. 1:**
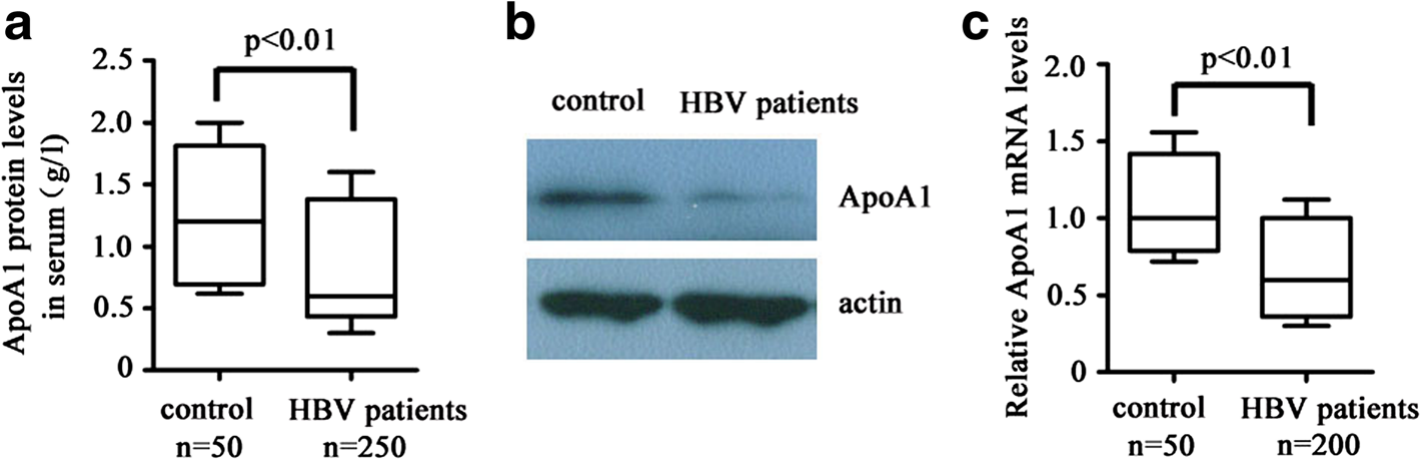
ApoA1 expression was significantly decreased in CHB patients. **a** plasma ApoA1 levels were performed by ELISA in 250 CHB patients corresponding 50 healthy control. **b** and **c** ApoA1 protein levels in live tissue sections were detected by Western blot wherase ApoA1 mRNA levels from 200 CHB patients and 50 healthy controls were analyzed by RT-PCR. Results of the Real-time PCR were normalized to an endogenous control GAPDH. The ApoA1 mRNA levels in healthy controls were arbitrarily set as 1.0. Error bars are means ± standard deviation (SD). Data are presented as the mean ± SD from three independent experiments. *p < 0.05 and **P < 0.01 compared with mock

Although ApoA1 expression has been shown to be specifically suppressed in CHB patients corresponding healthy controls [1], the evidences remain insufficient enough. To highlight this question, we first detected plasma ApoA1 expression from 250 CHB patients and 50 healthy controls by ELISA. As can be seen in Fig. 1a, plasma ApoA1 expression was significantly decreased by 39.9 % in CHB patients corresponding healthy controls. To further determine the changes of ApoA1 expression in lives of CHB patients, we detected ApoA1 protein and mRNA levels by Western blot and RT-PCR, respectively. As demonstrated in Fig. 1b and c, ApoA1 protein and mRNA levels were decreased dramatically in CHB patients compared with healthy controls.

### HBV induced down-regulation of ApoA1

**Fig. 2:**
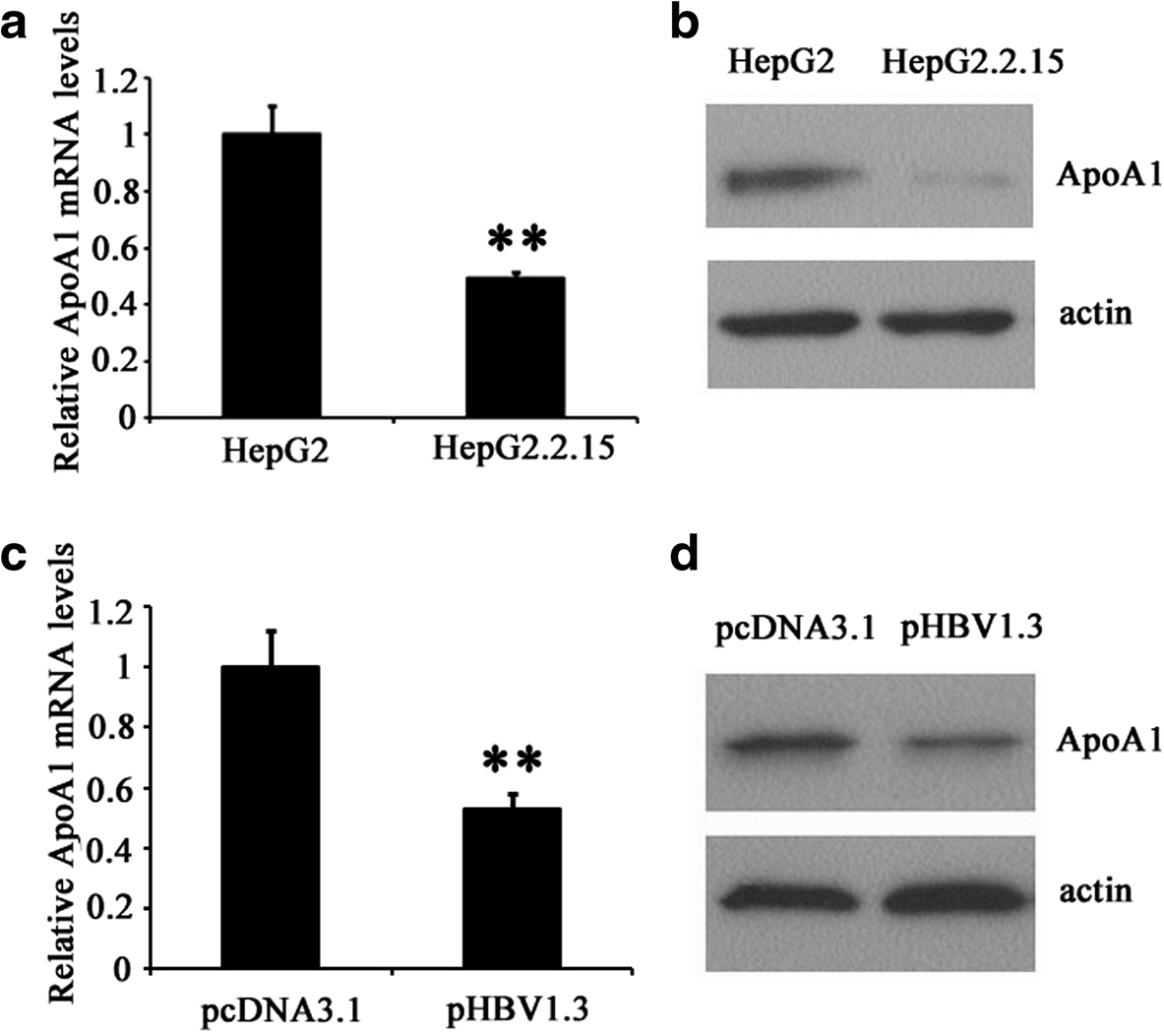
Suppression of ApoA1 expression by HBV. **a** and **b** ApoA1 mRNA and protein levels were detected by RT-PCR and Western blot in HepG2.2.15 corresponding HepG2 cell lines. **c** and **d** HepG2 cells were transfected with 2 μg pHBV1.3 plasmid or 2 μg pCDNA3.1 as control, ApoA1 mRNA and protein levels were detected at 48 h after transfection. Data are presented as the mean ± SD from three independent experiments. *p < 0.05 and **P < 0.01 compared with mock

Next, we explored whether the expression of ApoA1 was affected by HBV in hepatoma cells, HepG2 and HepG2.2.15 cells were selected for testing whether ApoA1 was subject to regulation by HBV because HepG2.2.15 cells stably produce HBV virus and derived from HepG2 cell lines. As can be seen in Fig. 2a, ApoA1 mRNA levels were significantly decreased by 50.6 % in HepG2.2.15 corresponding HepG2 cells. Moreover, ApoA1 protein levels detected by Western blot were dramatically reduced (Fig. 2b). To highlight whether the suppression of ApoA1 due to HBV expression, HepG2 cells were transfected with 2 μg pHBV1.3 plasmid or 2 μg pCDNA3.1 as control, expression of ApoA1 mRNA and protein levels was apparently decreased at 48 h after pHBV1.3 transfection. These results suggested HBV can inhibit ApoA1 mRNA and protein levels in hepatoma cells.

### ApoA1 expression was suppressed mainly through ApoA1 promotor hypermethylation by HBV

**Fig. 3:**
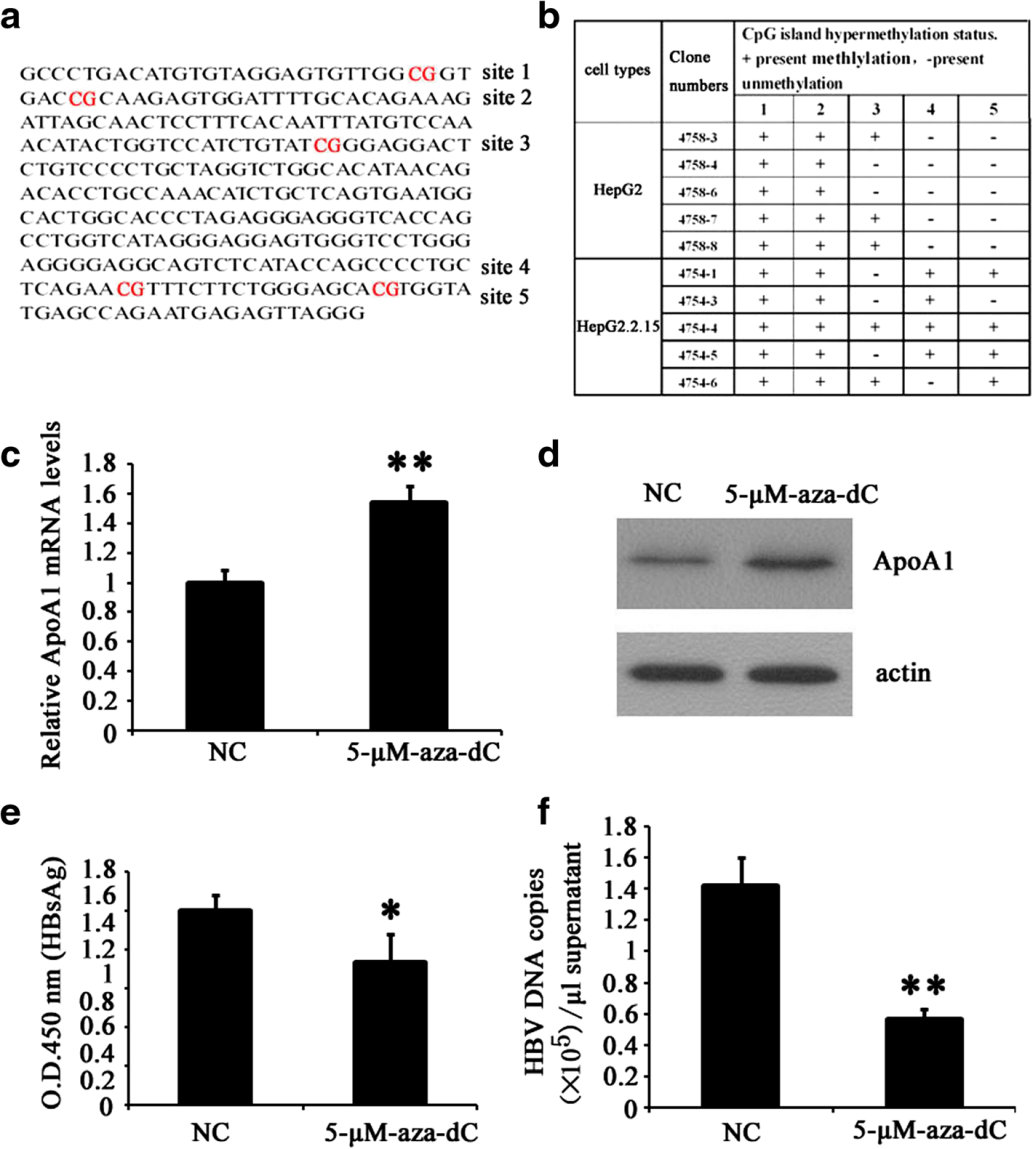
ApoA1 expression was suppressed by DNA methyltransferase inhibitor 5-aza-dC. **a** and **b** 5 CpG islands including two methylation CpG status in ApoA1 promotor were listed (**a**), the detection results of the 5 CpG islands methylation status were shown (**b**). HepG2.2.15 cells treated with 5 μM 5-aza-dC 48 h, ApoA1 mRNA and protein levels were detected by RT-PCR and Western blot respectively (**c** and **d**). Secretion of HBsAg and HBV particles in the supernatant were detected by ELISA and RT- PCR respectivly (**e** and **f**). Data are presented as the mean ± SD from three independent experiments. *p < 0.05 and **P < 0.01 compared with mock

According to previous studies, ApoA1 expression and promoter activities were both inhibited by HBV [1]. To further demonstrate the underlying molecular mechanism, we surveyed the CpG island hypermethylation status in ApoA1 promotor by MSP in HepG2.2.15 corresponding HepG2 cells, the data showed that there were 11 CpG islands found in ApoA1 promotor while two CpG islands were hypermethylated. The position of 5 CpG islands including two hypermethylation status (sites 4 and sites 5) were shown in Fig. 3a (other data not shown), the methylation results of the 5 CpG islands were shown in Fig. 3b. To illustrate whether ApoA1 expression was affected by ApoA1 promotor hypermethylation, HepG2.2.15 cells were treated with 5 μM DNA methyltransferase inhibitor 5-aza-dC for 48 h. We chose to use 5 μM 5-aza-dC for the experiment because this concentration has been verified efficiently [16]. Then, ApoA1 mRNA and protein levels were detected by RT-PCR and Western blot respectively. As shown in Fig. 3c, ApoA1 mRNA levels with 5-aza-dC treatment were apparently elevated by 53.5 % corresponding negative control. Furthermore, ApoA1 protein levels were also apparently increased (Fig. 3d). Moreover, secretion of HBsAg and HBV paticles in the supernatant of HepG2.2.15 cells treated with 5 μM 5-aza-dC were apparently decreased by 26.8 % and 60.4 % respectivly (Fig. 3e and f). Our results demonstrated suppression of ApoA1 expression may be due to its promotor hypermethylation by HBV.

### Decreased HBV expression in HepG2.2.15 cells with 5-aza-dC treatment via up-regulation of ApoA1 expression

**Fig. 4:**
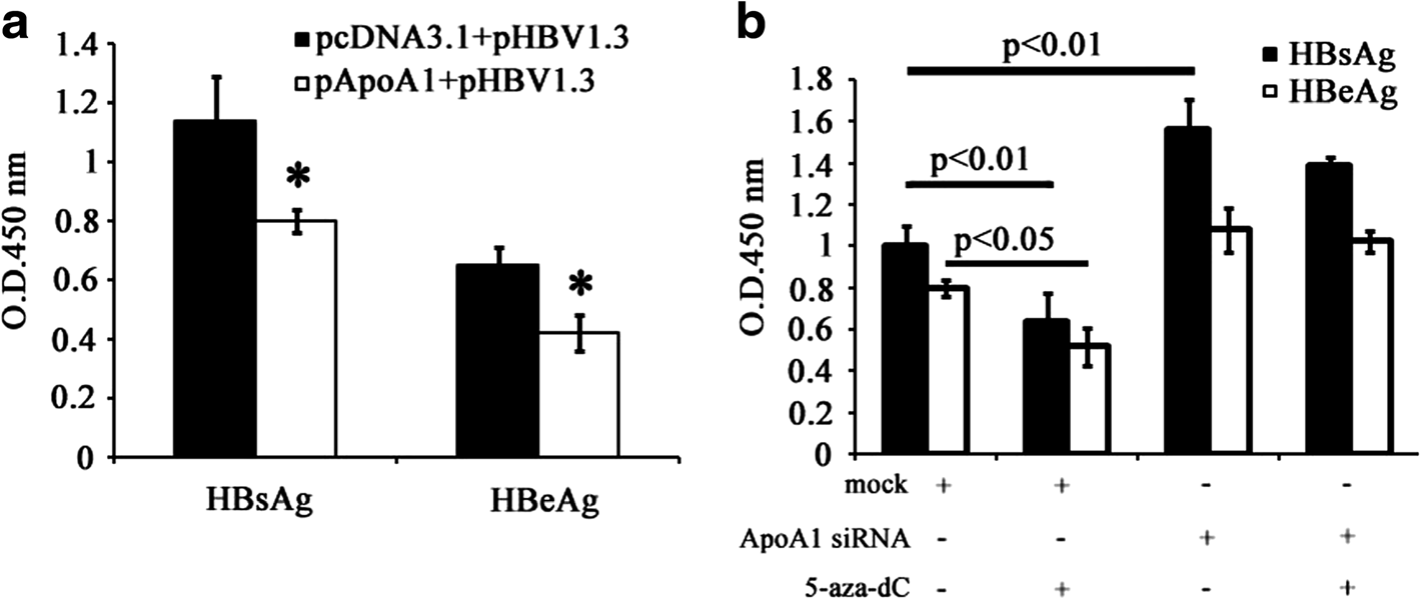
Decreased HBV expression with 5-aza-dC treatment via up-regulation of ApoA1 expression. **a** ApoA1 overexpression inversely suppressed HBV expression. HepG2 cells were cotransfected with 1 μg pApoA1 plasmid and 1 μg pHBV1.3. Sectetion of HBsAg and HBeAg were analyzed by ELISA at 48 h after transfection. **b** The inhibitory effect of 5-aza-dC on HBV expression was completely abolished by blocking 5-aza-dC-induced up-regulation of ApoA1 using RNAi. HepG2.2.15 cells were treated with 5 μM 5-aza-dC plus 50 μM ApoA1 siRNA or negative control, expression of HBsAg and HBeAg in the supernatant were analyzed by ELISA at 48 h. Data are presented as the mean ± SD from three independent experiments. *p < 0.05 and **P < 0.01 compared with mock

To confirm the functional role of ApoA1 in the life cycle of HBV, HepG2 cells were cotransfected with 1 μg pApoA1 plasmid and 1 μg pHBV1.3. Secretion of HBsAg and HBeAg were analyzed by ELISA at 48 h after transfection (Fig. 4a). Results showed that over expression of ApoA1 remarkably reduced the secretion of HBsAg by 30 % and HBeAg by 35.4 %. In conclusion, our results have shown 5-aza-dC treatment in HepG2.2.15 cells increased ApoA1 expression and decreased HBV production while ApoA1 overexpression suppressed HBV expression in HepG2 cells (Fig. 3e-f and Fig. 4a). To explore whether decreased HBV expression in 5-aza-dC treated HepG2.2.15 cells via up-regulation of ApoA1 levels, HepG2.2.15 cells were treated with ApoA1 siRNA. As indicated in Fig. 4b, analysis of HBsAg and HBeAg in the supernatant by ELISA revealed approximately equal amount of HBsAg and HBeAg between 5-aza-dC plus ApoA1 siRNA treated cells and negative control. Notably, the inhibitory effect of 5-aza-dC on HBV expression was completely abolished by blocking 5-aza-dC-induced up-regulation of ApoA1 mRNA using RNAi (*P >* 0.05).

## Discussion

Most cases of HCC are HBV-related in china. Abnormal liver functions are correlated with altered liver protein profiles, which may play important roles in the development and prognosis of liver injury [17]. The protein expression profiles have been detected with CHB and HBV-related cirrhosis patients [9]. ApoA1 was paid more attention because of the altered expression and migration pattern. However, contradictory results about the levels of plasma proteins in various liver diseases were obtained. Earlier studies reported that plasma proteins such as haptoglobin, α1-antitrypsin, and ApoA1 were suppressed in CHB patients while other studies showed different results [9, 18]. To further confirm the variation trends of ApoA1, our results suggested plasma ApoA1 as well as ApoA1 expression in CHB patients were decreased corresponding healthy controls in vivo. Moreover, expression of ApoA1 mRNA and protein levels was inhibited by HBV in the dose dependent manner in hepatoma cells.

The molecular mechanism of ApoA1 down-regulated by HBV was further explored. In our previous study, ApoA1 was confirmed as a HBx-interacting protein and its lipid-binding ability was impaired by HBx [12]. Furthermore, the luciferase activities of ApoA1 promotor was also down regulated by HBV in hepatoma cells [1]. However, the signal pathway of the regulation of HBV on ApoA1 expression and its exact mechanism still waits further investigation. We found 11 CpG islands in ApoA1 promotor and surveyed the CpG island hypermethylation status by MSP in HepG2.2.15 cells corresponding HepG2 cells. Two CpG island hypermethylation status were detected in ApoA1 promotor in HepG2.2.15 cells (Fig. 3b). It is well known that DNA methylation is governed by the interaction of DNA methyltransferases (DNMT) whose activities are inhibited by DNA methyltransferases inhibitor 5-aza-dC [19, 20]. A recent study demonstrated the mRNA levels of DNMT1, DNMT3A and DNMT3B in HBV-associated tissues were significantly higher than in the non-HBV-associated tissues, which may lead to hypermethylation/inactivation of p16 [21]. To determine whether the CpG island DNA hypermethylation of ApoA1 might be related with HBV infection, HepG2 cells, transfected with 2 μg pHBV1.3, were treated with DNA methyltransferase inhibitor 5-aza-dC, the inhibitory effect of HBV on ApoA1 expression was completely abolished, indicating suppression of ApoA1 by HBV may be through hypermethylating ApoA1 promotor via up-regulation of DNMT1, DNMT3A and DNMT3B [21, 22].

To further confirm the suppressed expression of HBV induced by 5-aza-dC due to up-regulation of ApoA1 mRNA and protein levels, we found that decreased HBV expression was completely abolished by ApoA1 knockdown (Fig. 4b). Furthermore, secretion of HBsAg and HBeAg was inhibited by ApoA1 overexpression, indicating ApoA1 inversly enhanced HBV expression, which was in line with our previous study [12]. The potential mechanism was that ApoA1 suppressed HBV expression via inhibiting cellular cholesterol levels required for HBV infection and escape from host cellular membrane.

## Conclusions

In conclusion, our current study herein explored the mechanism of ApoA1 down-regulated by HBV whereas suppressed ApoA1 expression could further promote HBV expression, which contributed to reveal the pathogenesis of CHB. In addition, the overexpression of ApoA1 would inhibit HBV expression, providing the theoretical basis for clinical diagnosis and treatment of CHB.
